# The simplified tailor-made workflows for a 3D slicer-based craniofacial implant design

**DOI:** 10.1038/s41598-023-30117-w

**Published:** 2023-02-17

**Authors:** Suchada Tantisatirapong, Sarunyapong Khunakornpattanakarn, Thanyakarn Suesatsakul, Amaraporn Boonpratatong, Itsara Benjamin, Somprasong Tongmeesee, Tanasit Kangkorn, Theerasak Chanwimalueang

**Affiliations:** 1 Department of Biomedical Engineering, Faculty of Engineering, Srinakharinwirot University, Nakhon Nayok, 26120 Thailand; 2 Division of Plastic and Reconstructive Surgery, Department of Surgery, Chonburi Hospital, Chonburi, 20000 Thailand

**Keywords:** Biomedical engineering, Bone imaging

## Abstract

A specific design of craniofacial implant model is vital and urgent for patients with traumatic head injury. The mirror technique is commonly used for modeling these implants, but it requires the presence of a healthy skull region opposite to the defect. To address this limitation, we propose three processing workflows for modeling craniofacial implants: the mirror method, the baffle planner, and the baffle-based mirror guideline. These workflows are based on extension modules on the 3D Slicer platform and were developed to simplify the modeling process for a variety of craniofacial scenarios. To evaluate the effectiveness of these proposed workflows, we investigated craniofacial CT datasets collected from four accidental cases. The designed implant models were created using the three proposed workflows and compared to reference models created by an experienced neurosurgeon. The spatial properties of the models were evaluated using performance metrics. Our results show that the mirror method is suitable for cases where a healthy skull region can be completely reflected to the defect region. The baffle planner module offers a flexible prototype model that can be fit independently to any defect location, but it requires customized refinement of contour and thickness to fill the missing region seamlessly and relies on the user's experience and expertise. The proposed baffle-based mirror guideline method strengthens the baffle planner method by tracing the mirrored surface. Overall, our study suggests that the three proposed workflows for craniofacial implant modeling simplify the process and can be practically applied to a variety of craniofacial scenarios. These findings have the potential to improve the care of patients with traumatic head injuries and could be used by neurosurgeons and other medical professionals.

## Introduction

Designing and manufacturing craniofacial implants based on CT/MRI images has been commercially available since 2003^1^. Although the technology has brought the well-recognized benefits, the productivity and accessibility are limited due to complex procedures, time consumption and technology costs^1,2^. Such technology affects survival rates of craniofacial bone injuries which are mostly due to road and occupational accidences. A number of such cases has significantly increased in developing countries especially Asia where industrial parks have been established^3^.

Craniofacial trauma is a common occurrence in motorcycle accidents, and many of these cases involve individuals who use motorcycles as their daily means of transportation^3–5^. As a result, there have been numerous cases of craniofacial surgery, but there is a shortage of craniofacial surgeons and facilities to adequately manage these cases^6^.

Earlier, the surgical procedure for managing skull defects was complicated as a prototype of implant must be designed and formed by hand^7^. Customizing such implant is time consumption and needs experts who can manually approximate defect shape based on underlying skull structure appearing on 2-dimensional x-ray or CT images. Moreover, the implant must be instantaneously refined at a pre-operation or during intraoperation. This traditional process requires expertise which can be labor-intensive and cost insufficient as well as results in complex operations and poor aesthetic outcome^1,2,8^. Recently, advances in Computer Aided Design (CAD) and Computer-Aided Manufacturing (CAM) technologies have led to a precise implant design platform for which a combination of 3D anatomical reconstruction software and 3D printing system has been employed. The commercial software has been developed as a crafting tool for customizing cranioplasty implants, such as MIMICS (Materialise NV, Belgium), Geomagic (3D Systems, South Carolina) Biobuild and 3D doctor^9–11^. This software allows patients to preview and examine the expected outcome of the implant operation^3^created using the three techni. Several studies have been proposed to design patient-specific craniofacial implant models^11–21^.

The development of a computer-based craniofacial implant involves in 2D and 3D image processing techniques. Estimation and reconstruction of skull detects, or missing parts have been widely studied. Abdullah et al.^11^ presented the process of creating a cranial implant using the open-source MITK software. The ten CT scan-based implant models were compared to the underlying models created from the commercial 3-Matic software. The shape-based interpolation method was applied to the segmented slices of the obtained CT data. Such method shows accurate results compared to the human-based design for patient-specific cranial implants. Many studies have proposed a mirror image reconstruction technique for designing implant model^12,13,19,22^. Moiduddin et al.^12^ investigated a replication technique based on mirroring healthy skull along the midsagittal plane to the missing bone segment. To handle the gaps and discontinuous surfaces, the process of joining and wrapping was implemented. This study shows that the skull thickness of a personalized cranial implant is accurately identical to the thickness of the skull. Saldarriaga et al.^13^ demonstrated the use of a CAD/CAM program to design cranial implants based on the mirror-image function. The intact contralateral anatomy of patients with traumatic injuries was employed as templates and used for mirroring the surface area of defects. Their findings revealed that the mirror surface reconstruction obtained from such templates was acceptable. However, the technique was unable to applied for a case where defect located on the area around the midsagittal line effectively. Senck et al^14^ introduced a combination of the virtual anthropology tools and the geometric morphometric methodologies used for reconstructing severely injured crania (i.e. the area around the midsagittal cranial plane is partially or entirely missing). Thin Plate Spline (TPS) interpolation was used to estimate the missing data after applied the mirrored image. The TPS shows a reduction in bending energy of the thin plane spline between the reference and the target. Min et al.^15^ proposed a design of craniofacial implant based on mechanical topology. Load, displacement, stress, and boundary conditions were formed as an objective function for finite element analysis. The proposed method indicates a reduction in volume of implant (− 23%) resulting in good mechanical performance. Murphy et al.^16^ developed a computer-assisted single-stage method for cranioplasty during pre-operation or intraoperation. The surgeon was guided to adapt the oversized implant to match the resected area by projecting the outline of the resected bone onto the actual implant. This study demonstrated precise implant fit with small area of defect necessary for implant modification. However, if the size of the implant is too large, this procedure is unable to function effectively.

Gill Kaur et al.^17^ used the 3D Slicer software (https://www.slicer.org/) to design an implant model based on skin surfacing and mesh refining. They applied the mirroring approach to split the right frontal cranium and then reflected as a patch to the defect area. Boolean operations were applied to remove the overlapping volume and optimize the implant model. Woon-Man et al.^18^ explicated the development of CAD/CAM techniques used in the reconstruction of skull defects. The CT scan data were obtained to create a deficient skull model and then employed for reconstructing cranial defects. The study also informed that skull models can be reconstructed using a variety of commercial programs and libraries, for examples Open-Source Computer Vision (OpenCV), Open Graphic Library (OpenGL) OBJ Viewer and GLC Player. Mian Hammad et al.^19^ performed the mirror image reconstruction technique to create a personalized cranial implant. The left defective portion was replaced with the healthy right side of the skull model, preserving anatomical symmetry. To remove the gaps in the model, merging and wrapping procedures were conducted. Taper screw holes were made for implant placement and stability. Jianning et al.^20^ proposed an automatic design workflow for cranial implant. The workflow is based on deep-learning networks. Artificial defects were obtained from 24 patients’ CT scan datasets. The extracted data were trained and validated using an automatic prediction as either the complete skulls or the implants. The shape and location of artificial defects were simplified and placed on specific areas. However, the artificial defects were far from the actual craniotomy defects whereby their location can appear randomly with a complex defect structure. The artificial network systems still required improvement in terms of training to be more generic.

As aforementioned reviews, the numerical based implant design requires supervision of experienced practitioners, resources in medical organization, to guarantee anatomical and surgical compatibility^10^. One of the practical solutions to this bottleneck problem, therefore, is to propose a simplified workflow for cranioplasty implant design. This aims to comfort neurosurgeons or experts to individually create craniofacial implants by themselves. In our study, we investigated the three modelling-based workflows: (i) mirror, (ii) baffle planner, and (iii) baffle-based mirror guideline (a new approach proposed in this study). These workflows are based on an implementation on the freeware “3D Slicer” pervasively used by medical support personnel such as medical engineers, researchers, and scientists. The three modelling workflows were investigated and evaluated against the references (the implant models designed and customized by the experienced neurosurgeon and used as the standard models).

## Materials

**Figure 1:**
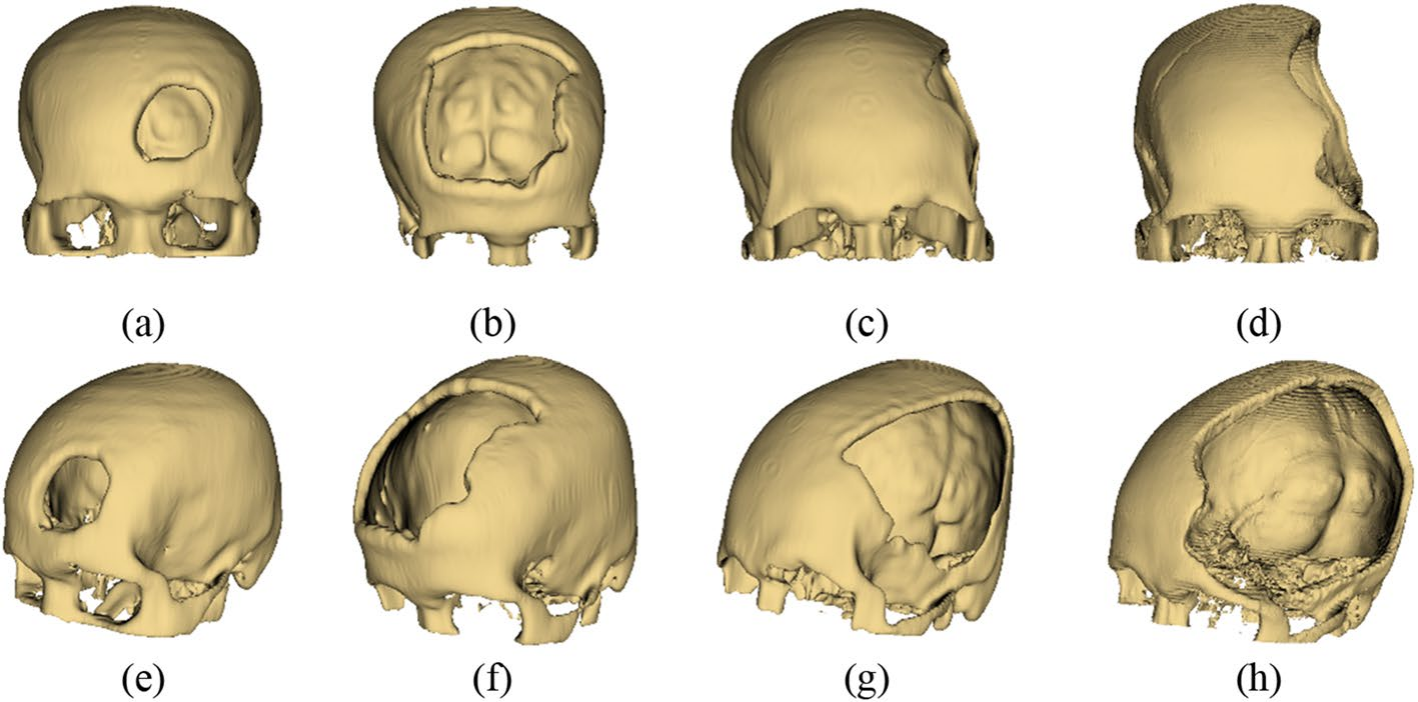
Frontal and perspective views of the 3D reconstructed skull models of the four patients whose defects appear on four locations. The top row (**a**) and (**b**) illustrates the frontal defect cases while (**c**) and (**d**) depicts parieto-temporal defect cases. The bottom row (**e**) to (**h**) represents the perspective views of cases (**a**) to (**d**). Note that the four cases were named as case A, B, C and D corresponding to (**a**), (**b**), (**c**) and (**d**) respectively.

We obtained the four different CT scan datasets from four patients who had head trauma caused by severe road accidents. All patients were admitted at the Chonburi hospital, Thailand and underwent craniofacial surgery. The craniofacial section of the four subjects was only acquired from CT scanner (GE Medical Systems and Toshiba) and recorded in Digital Imaging and Communications in Medicine (DICOM) format, which has a matrix size of 512 × 512. We reconstructed the four datasets using the 3D Slicer software (https://www.slicer.org/)^23^ and then extracted only skull density to illustrate size and location of particular case as depicted in Fig. 1. It is noted that the 3D slicer allows users to develop algorithms and used as extension modules for any specific purposes, such as enhanced filtering. The cases represent good scenarios for determining an effect of sizes and locations of defect when modelling implants using our proposed workflows (details are described in “Methods”). We named the four datasets as case A, B, C and D, as described in Table 1.

**Table 1:** Detail of craniofacial defects of four patients used in this study.

Case	Gender	Age	CT scanner	Defect description
A	Male	65	GE Medical Systems	Defect appears on the frontal bone but mainly on the left side of the frontal bone and some slight area across the right side of the frontal bone (based on the midsagittal plane)
B	Female	67	GE Medical Systems	Defect appears largely across left and right sides on the frontal bones
C	Male	26	GE Medical Systems	Defect appears mainly on the left temporal with a conjunction of the left side of the parietal bone
D	Female	46	Toshiba	Defect appears mainly on the left temporal with a conjunction of the left side of the parietal bone but with larger defect covering superior and anterior area compared to defect area of case C

It should be mentioned that the size of each case was measured by the reference implant models. These reference models were created by an experienced neurosurgeon who has worked at Chonburi Hospital, Thailand. The reference models were manually designed and customized using a combination of three freeware: “3D Slicer”, “Meshmixer” (Autodesk Meshmixer (RRID:SCR_015736)), and “Blender”^24^, which is described in “Appendix”. The version of 3D Slicer, Meshmixer and Blender are 4.10, 3.5 and 2.75 respectively.

## Methods

This study was approved by the Institutional Review Board, Chonburi Hospital, with the reference number 72/65/O/h3 and all participants provided informed consent. This study was performed in accordance with relevant guidelines and regulations.

**Figure 2:**
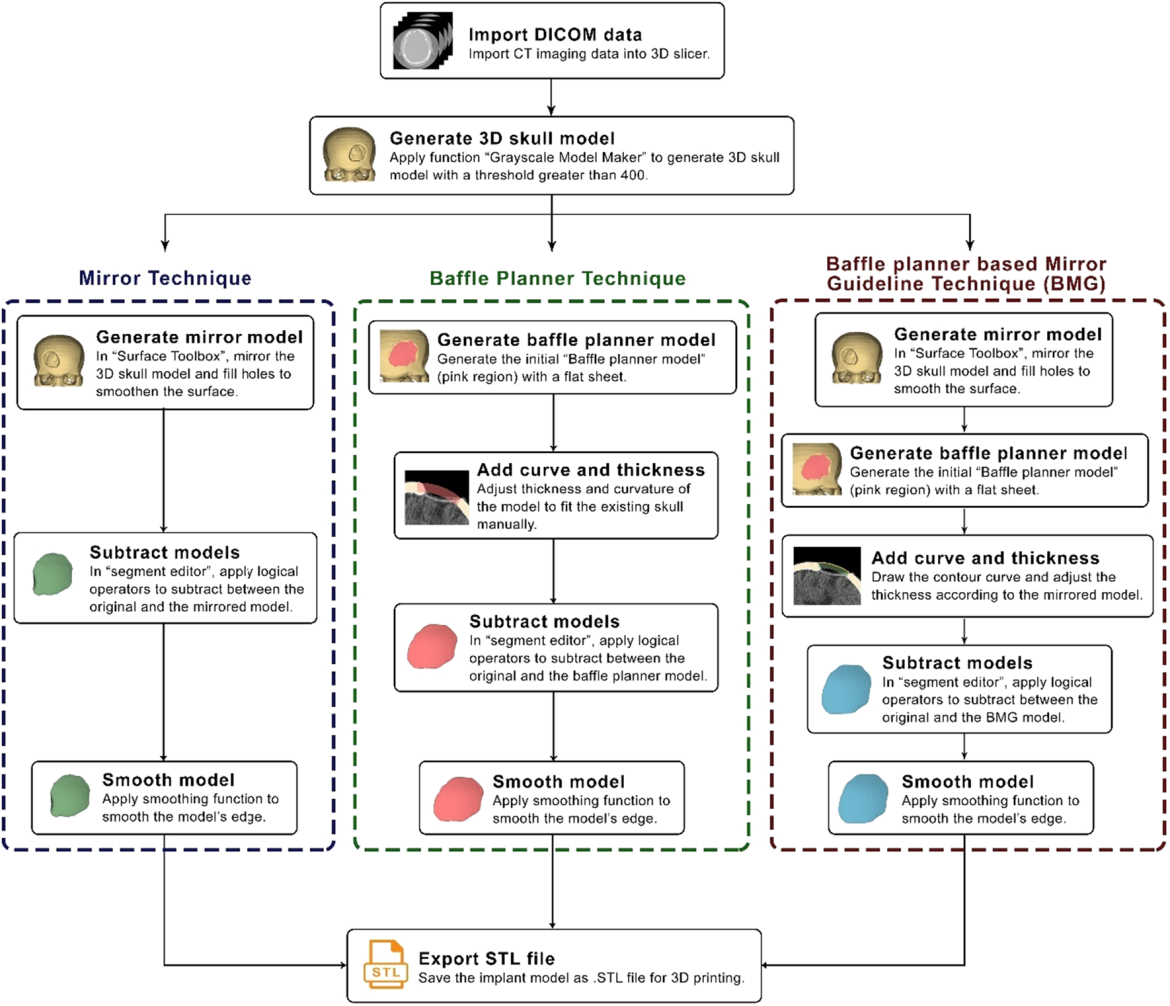
Processing workflows of the mirror, baffle planner and baffle planner module-based modelling.

In this study, we proposed three processing workflows to modelling craniofacial implants: (i) mirror (ii) baffle planner and (iii) baffle-based mirror guideline (BMG) as depicted in Fig. 2. The three workflows were conducted under 3D Slicer software version 4.11.20210226 and described in the following sections.

### Mirror based modelling workflow

**Figure 3:**
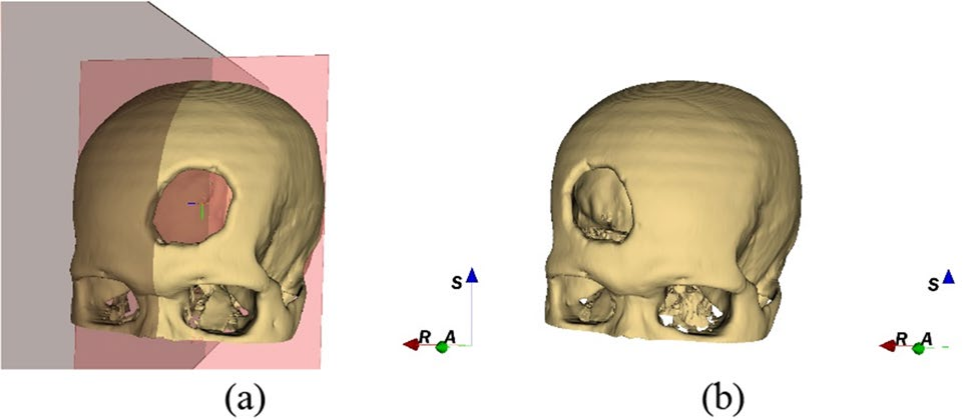
Mirroring the skull defect area along the midsagittal plane (**a**) create the midline on the sagittal plane (**b**) mirror the surface between the left and right sides of skull to obtain a mirrored whole skull. S, R, A stand for superior, right, and anterior.

The mirror-based technique is relied on an assumption that the left and right sides of skull centered by the midsagittal plane are approximately symmetrical. Therefore, any missing part on the left or right side of skull can be replaced by flipping an undetected side to the defected side along the midsagittal plane, as illustrated in Fig. 3. In 3D Slicer, the mirrored model can be replicated using a linear transformation. Given the original coordinate matrix M, the mirror matrix *M*′ is calculated as Eq. (1).

1 $$M^{\prime}=\left[\begin{array}{ccc}-1& 0& 0\\ 0& 1& 0\\ 0& 0& 1\end{array}\right]M$$

**Figure 4:**
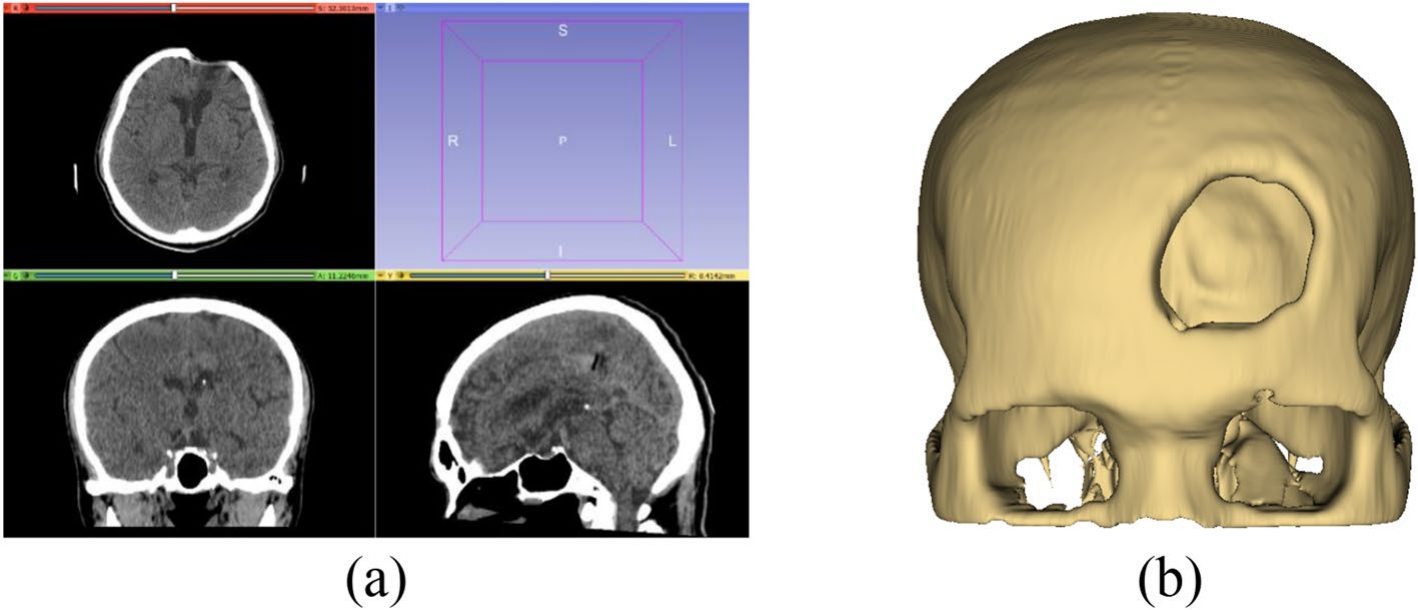
Mirror based modelling (**a**) import DICOM data and (**b**) reconstruct 3D model.

**Figure 5:**
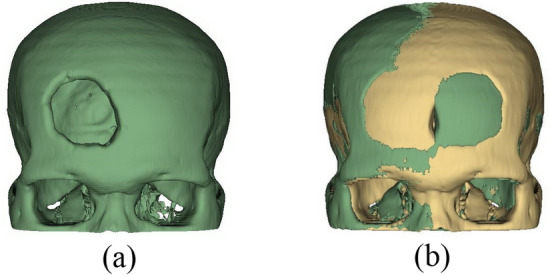
Mirror based modelling creates (**a**) the mirror model and (**b**) the overlayed model between the original model and its mirror version.

**Figure 6:**
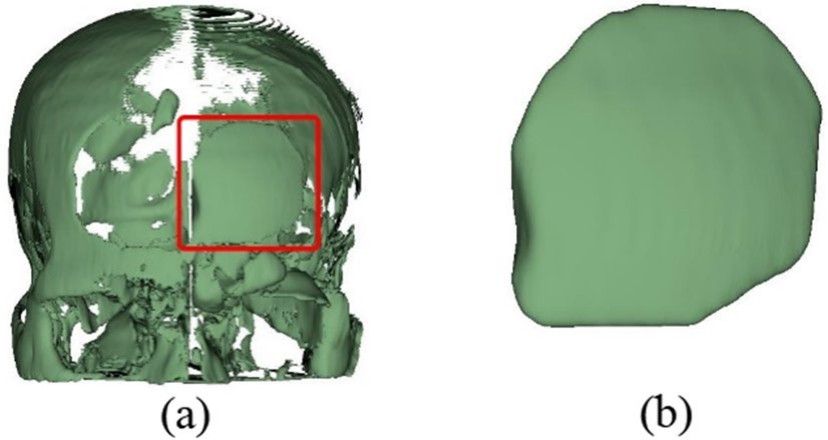
Mirror based modelling: (**a**) subtract between the mirror and reference models, (**b**) remove residual and smoothen the model.

We proposed five steps to create an implant model based on the mirror technique implemented on the 3D Slicer as follows:

1. Import a CT DICOM dataset into 3D Slicer and use the ‘Grayscale Model Maker’ module to reconstruct the dataset as a 3D model on a sagittal plane. After reconstructing, a threshold of the Hounsfield scale of CT numbers is set to not less than 400 to extract skull (bone intensity)^25^, so that any soft tissues and physiological fluid are eliminated as shown in Fig. 4. In the case of orbital implant, the threshold can be set to 100–200 to preserve the lower intensity of orbital bone structure. This extracts a 3D skull model as a prototype for the next step.
2. Duplicate the 3D skull model to create its mirror version using the ‘Surface Toolbox’ along the x axis (Fig. 5a). Then the missing volume of the mirror model is filled using morphological operator under the function ‘Flood filling’ with value of 1000 (a maximum hole size).
3. After saving the original model and its mirrored 3D version, in the segmentation module, apply a subtraction operator (selected under the menu ‘Logical operators’) to delete the overlapping volumes between the two models (Fig. 5b).
4. To refine the implant model resulting from step 3, apply the ‘Scissors’ tool to remove residuals and apply ‘smoothing’ tool (median based smoothing method with kernel size of 2 mm, which is 5 × 5 × 1 pixel) to smoothen the surface (Fig. 6a).
5. After completing the refinement, save the implant as STL (Standard Tessellation Language) file format for 3D printing (Fig. 6b).

### Baffle planner module based modelling workflow

The baffle planner is a module under the ‘SlicerHeart’ extension, which contains a variety of modules useful for cardiac analysis and intervention planning and guidance. The baffle planner module is able to generate an infinitely thin sheet as an open surface, or a closed surface with specified thickness. This technique was initially developed for modeling intracardiac baffle in double-outlet right ventricle^26^. Furthermore, based on flexibility of baffle sheet, the approach can be used for enhancing any smooth 3D surface or regenerating some anatomical structure. The baffle planner module allows users to define a closed boundary of the defect skull region and create a 3D baffle patch. The thin-plate spline inter- and extrapolation is used to estimate a curved surface with minimal bending energy.

In this study, we performed various functions in the 3D Slicer to design the four craniofacial implant models. The processing workflow is described as follows.

**Figure 7:**
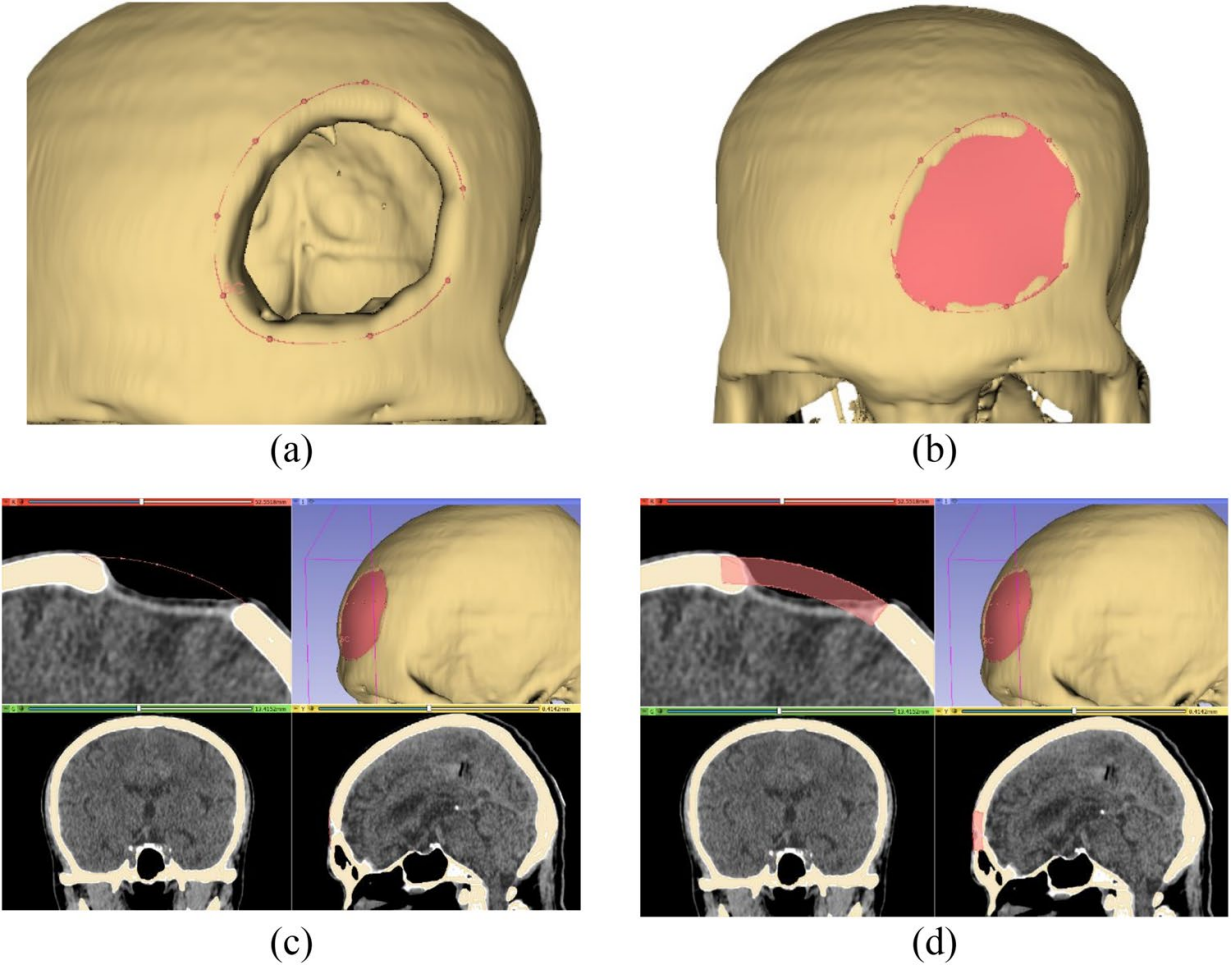
Baffle planner-based modelling: (**a**) marking points around the defect, (**b**) generating planner over defect, (**c**) and (**d**) defining curvature and thickness of the implant model.

**Figure 8:**
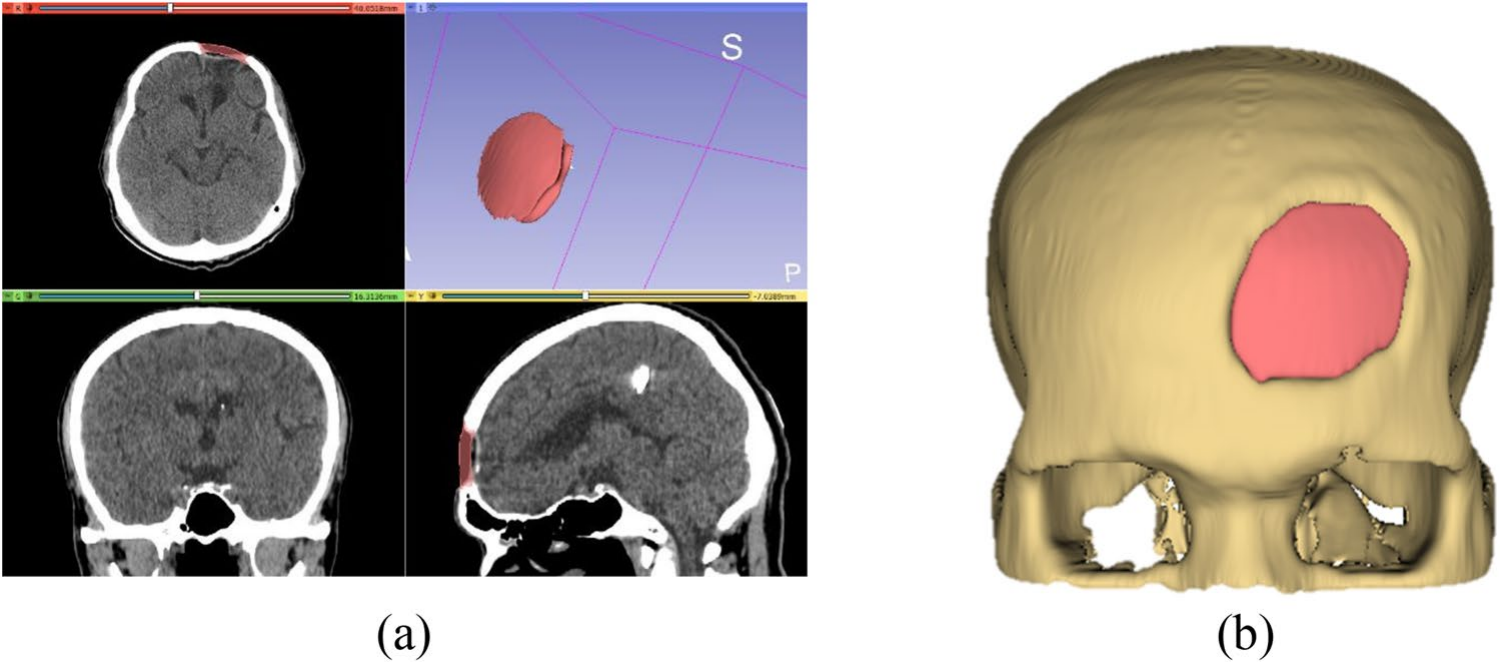
Baffle planner-based modelling: (**a**) model enhancement, (**b**) final implant model.

1. Import a CT DICOM dataset and reconstruct the 3D skull model as the procedure conducted in the mirror-based modelling workflow.
2. Switch to baffle planner module. Mark points to create a closed boundary of the defect area (Fig. 7a,b). Such delineation is essential for limiting growth area expanded from the baffle sheet prototype. Subsequently, create the baffle model under the menu ‘Baffle model’ and select ‘Create new Model’.
3. Adjust the curvature by manually relocating the marked points and set the skull thickness by adjusting the score bar, as shown in Fig. 7c,d.
4. In the “segment editor”, apply logical operator to subtract between the original and the baffle planner model to remove unwanted skull, Fig. 8a. Apply median based smoothing method with kernel size of 2 mm, which is 5 × 5 × 1 pixel to polish the implant surface (selected under the ‘Smoothing’ and ‘median function’ menu), Fig. 8b. The refined implant model is saved as .STL file for printing.

### A baffle based mirror guideline method (BMG)

**Figure 9:**
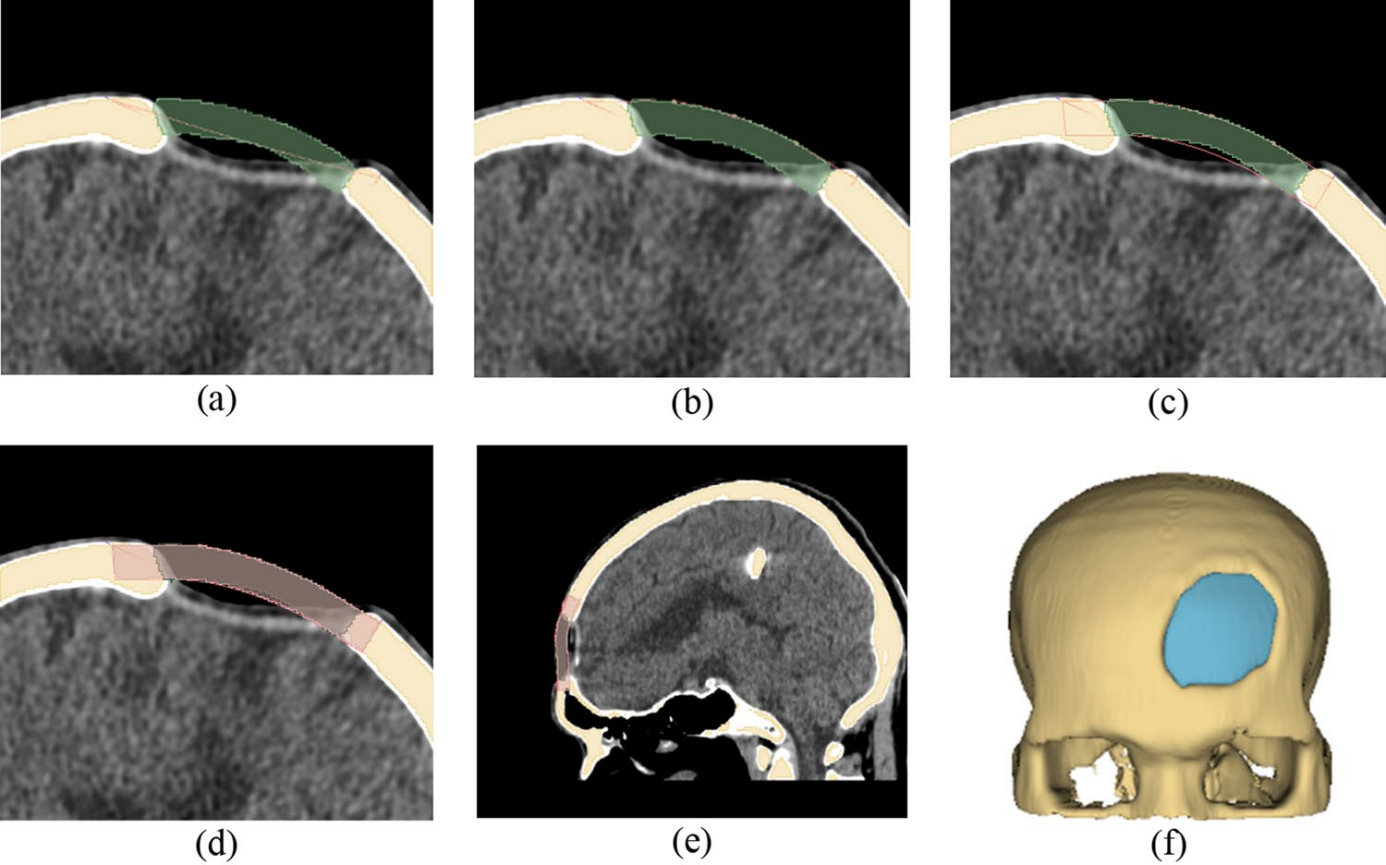
A Baffle based Mirror Guideline Method (BMG) (**a**) overlay the mirror implant model on the baffle planner layer (**b**) draw a curvature along the outer surface of the mirrored implant model (**c**) adjust model thickness to match the mirrored model (**d**) and (**e**) visualization of the combined mirror and baffle planner implant model on sagittal and coronal plane (**f**) final implant model.

We proposed the last workflow which utilized both the mirror and baffle planner methods. We named the workflow as the Baffle based Mirror Guideline (BMG). In this method, the mirror implant model was employed as a reference template, so only cases where area of defect is not largely located across the midline can be implemented. The first step of the workflow is to complete the mirror-based modelling and follows by the baffle planner method as shown in Fig. 9. A mirror-based guideline on the three planes: axial, sagittal and coronal was illustrated and used as a reference template. A user is able to manually adjust degrees of curvature of the baffle prototype at the any slices on such three planes (the mid slice of the defect on both planes is recommended). The aim of this workflow is to overcome the drawback of the baffle planner technique in terms of shape estimation whereby a user must approximate degree and distance of skull curve. The BMG processing workflow in the 3D Slicer is described as follows.

1. Import a CT DICOM dataset and use the mirror-based modeling workflow to reconstruct a 3D skull model and create an implant model. Save both the skull and implant models.
2. Load the original skull model and overlay the implant model on the defect volume. Using the baffle planner module, mark points to create a closed boundary following the curvature of the mirror model (refer to Fig. 9a,b).
3. Under the "Baffle Model" menu, select "Create New Model" and adjust the curvature by manually relocating the marked points. Set the skull thickness and curvature by adjusting the score bar as shown in Fig. 9c–e.
4. In the "Segment Editor," apply a logical operator to subtract the original skull model from the baffle planner model to remove the defect-free skull. Use a median-based smoothing method with a kernel size of 2 mm (5 × 5 × 1 pixels) to polish the implant surface (found under the "Smoothing" and "Median Function" menus). Save the refined implant model as an .STL file for printing, as shown in Fig. 9f.

## Performance metrics

**Figure 10:**
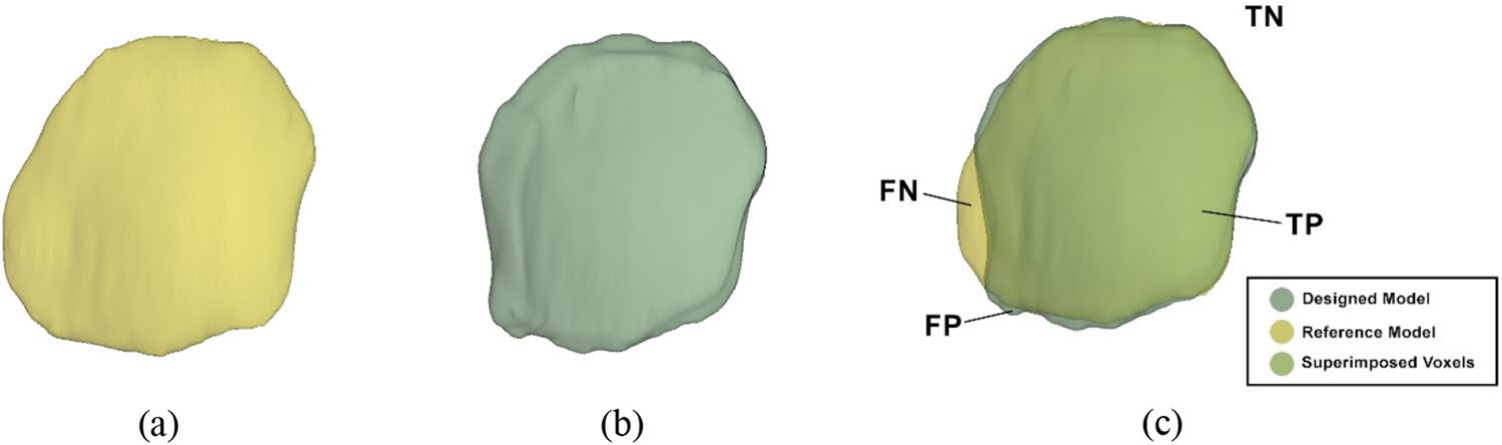
Implant model designed by (**a**) neurosurgeon, i.e., the reference defect model, (**b**) mirror-based method and (**c**) overlay of the designed and reference models. It should be noted that any voxels located outside both the designed and reference are defined as healthy skull.

Performance metrics were computed to indicate the degree of similarity of the designed implant models compared to the reference models. The designed and reference implant model were imported to the same window in 3D Slicer. The four evaluated parameters: true positive (TP), true negative (TN), false positive (FP), and false negative (FN) were computed based on superimposed voxel’s location between our designed models and the reference models (Fig. 10), as follows:

i. True Positive ($TP$): the voxels of the designed model that are correctly defined as defect.
ii. True Negative ($TN$): the voxels excluded from the designed and reference models that are correctly defined as healthy skull.
iii. False Negative ($FN$): the voxels of the reference model that are wrongly defined as healthy skull.
iv. False Positive ($FP$): the voxels of the designed model that are wrongly defined as defect.

The six indicators: accuracy, precision, sensitivity, specificity, Dice coefficients and Hausdorff distance, exemplify the spatial properties compared between the designed and reference models. These metrics can be computed based on the four evaluated values (TP, TN, FP, and FN), explained as follows:

Accuracy identifies the proportion of correct designed implant model compared to the reference model, as described in Eq. (2):

2 $$Accuracy= \frac{TP+TN }{TP+TN+FP+FN}$$

Precision measures the consistency of results, as described in Eq. (3):

3 $$Precision= \frac{TP }{TP+FP}$$

Sensitivity indicates the proportion of correct designed model, as described in Eq. (4):

4 $$Sensitivity= \frac{TP }{TP+FN}$$

Specificity determines the proportion of correct volume of healthy skull, as described in Eq. (5):

5 $$Specificity= \frac{TN}{TN+FP}$$

Dice coefficient defines the similarity between the designed and reference models, as described in Eq. (6):

6 $$Dice \; Coefficient= \frac{2{(V}_{1}\cap {V}_{2})}{{V}_{1}+ {V}_{2}}$$

where ${V}_{1}$ is the volume of the designed model and ${V}_{2}$ is the volume of the reference model.

The Hausdorff distance^27^ quantifies the degree of dissimilarity between two objects. The lower the value of the Hausdorff distance, the higher the degree of similarity between two objects. Note that when the Hausdorff distance equals zero, a perfect match occurs.

Given two finite point sets $A=\{{a}_{1},{a}_{2},{a}_{3},\dots ,{a}_{m}\}$ and $B=\{{b}_{1},{b}_{2},{b}_{3},\dots ,{b}_{n}\}$, the Hausdorff distance defines a distance between a point in A and B as the maximum of $h\left(A,B\right)$ and $h\left(B, A\right)$ as shown in Eq. (7):

7 $$H\left(A,B\right)=\mathrm{max}\left(h\left(A,B\right), h\left(B, A\right)\right)$$

where $d\left(a,b\right)$ is the Euclidean distance between $a$ and $b$. $h\left(A,B\right)=\underset{{a \in A}}{\mathrm{max}}\left(\underset{{b \in B}}{\mathrm{min}}d\left(a,b\right)\right)$ and $h\left(B, A\right) =\underset{b\in B}{\mathrm{max}}\left(\underset{a\in A}{\mathrm{min}}d\left(b,a\right)\right)$ are called the directed Hausdorff distance from $A$ to $B$ and from $B$ to $A$ respectively.

## Experimental results and discussions

**Figure 11:**
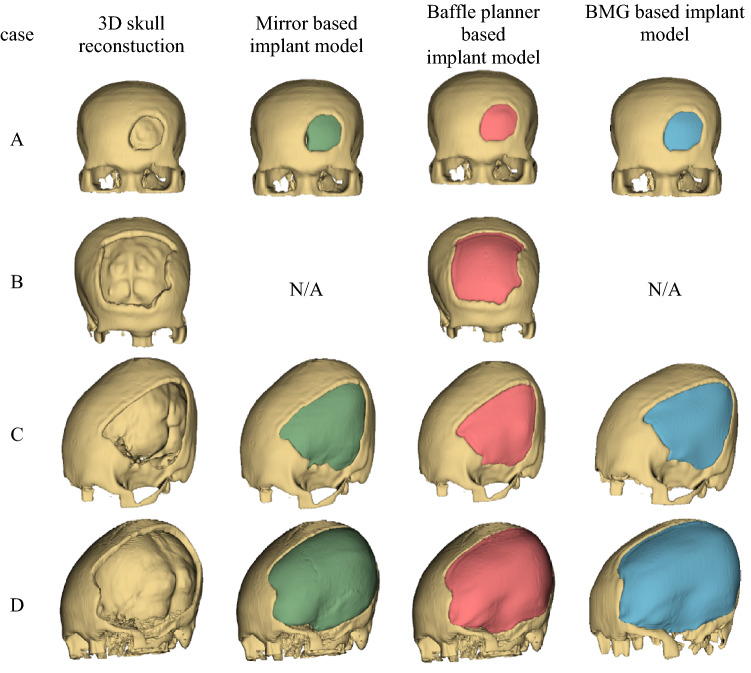
Customized craniofacial implants models for case (**A–D**) were created using the three techniques: (I) mirror, (ii) baffle planner and (iii) baffle-based mirror guideline. Note that the mirror and BMG techniques cannot create implant model for case B. N/A means that the implant model cannot be produced.

The craniofacial cases A, C and D were modelled using the mirror technique as depicted in Fig. 11. For case B, the implant was unable to be modelled since no healthy skull region can be mirrored (the large defect occurs on both left and right of the midsagittal line). However, for case A, the fracture appears mostly on the left hemisphere with a slight region overlapping the right hemisphere referred by the generated midsagittal line. This introduces asymmetrical problem to the mirror technique whereby some regions were generated randomly. The disadvantage of creating implant using the mirror method involves unsymmetrical skull and defect area across the midsagittal plane. As the left and right side of skull is not perfectly symmetrical, subtraction between the original and mirrored models results in residuals. Hence, a tool named ‘Scissors’ in the 3D Slicer must be applied to eliminate such residuals manually.

In terms of location, if the defect occurs on the midsagittal line, the mirror technique cannot reconstruct the missing volume locating between left and right hemisphere, as shown in Fig. 11. This drawback is resolved by the baffle planner technique which provides a more complete implant model than that generated using the mirror technique. This is because the center line of the skull is not required to model the implant. For cases A and B, the baffle planner method is able to model the implant for head trauma on frontal region effectively. However, manual adjustment, i.e., defining curvature and thickness of implant model, is required to create a precise implant model.

The surface area and volume of the designed implant models were computed using “Segment Statistics” module in 3D Slicer. The surface area and volume of designed implant models and reference models were compared in Table 2. It can be observed that in terms of volume error, the mirror technique is superior to the other two methods for case A, while the baffle planner technique outperforms the other two methods for cases C and D. When considering surface area error, the mirror, BMG, and baffle planner techniques show minimum error for case A, C and D respectively. Besides, the baffle planner method can only be implemented for case B, so it is not possible to compare the results of case B with results from other methods. We should note that the percentage of the surface area errors, and volume errors may not imply accuracy of the designed models correctly. Such errors represent only deviation in terms of physical quantities but not refer to appearance of voxels in a 3D coordinate. Therefore, the six-performance metrics regarding spatial properties were evaluated as described in performance metrics section.

**Table 2:** Comparison of surface and volume of the mirrored, baffle, BMG, and the reference models.

Case	Model	Surface area (mm^2^)	Volume (mm^3^)	Surface area error (%)	Volume error (%)
A	Reference	2912.0	5828.2	–	–
	Mirror	3164.9	6763.3	8.68	16.04
	Baffle	3205.0	7900.2	10.06	35.55
	BMG	3165.3	7460.9	8.70	28.01
B	Reference	14,968.9	35,374.8	–	–
	Mirror	N/A	N/A	–	–
	Baffle	15,325	41,430.8	2.38	17.12
	BMG	N/A	N/A	–	–
C	Reference	15,392.5	34,073.8	–	–
	Mirror	17,352.0	29,643.3	12.73	13.00
	Baffle	16,196.6	36,947.1	5.22	8.43
	BMG	15,624.6	29,045.5	1.51	14.76
D	Reference	35,564.6	93,314.6	–	–
	Mirror	37,970.9	116,653	6.77	25.01
	Baffle	37,197.6	104,737	4.59	12.24
	BMG	37,886.6	106,720	6.53	14.37

Surface area and volume errors of the designed models are computed against the reference models.

N/A means that the model is unable to be mirrored.

The six-performance metrics: accuracy, precision, sensitivity, specificity, Dice coefficients and Hausdorff distance were computed to evaluate the degrees of similarity between the designed and the reference models, as shown in Table 3. Before computing all the performance metrics, all implant models (.stl file) were translated to the center of origin using ‘surface toolbox’. Subsequently the performance metrics were computed using the module named ‘Segment Comparison’ in SlicerRT extension module in 3DSlicer software. It can be seen that the BMG method outperforms the other two methods for only case A (all metrics yield the highest values except precision). For case C, the mirror method outperforms the other two methods. This is because the skull thickness is not uniform. As a result, the mirror technique can preserve the width and bend of the implant model by duplicating the existing healthy skull. The baffle planner method can only create uniform skull thickness, while the mirror method generates symmetrical thickness based on a healthy skull side. For case D, the BMG method yields highest accuracy and specificity since the BMG method exploits the ability of both mirror and baffle planner methods to create the large missing defect. In addition, case B can solely be created by using the baffle planner method, so the result cannot be compared with the other two methods. This also recommends that the baffle planner method is suitable for the defect appearing around the midline.

**Table 3:** Comparison of designed model created by using mirror, baffle planner and BMG methods.

Case	Model	Accuracy (%)	Precision (%)	Sensitivity (%)	Specificity (%)	Dice coefficient	Hausdorff distance
A	Mirror	92.3	74.0	85.9	93.6	0.80	0.84
	Baffle	91.3	69.3	94.0	90.7	0.80	0.88
	BMG	92.2	73.3	93.1	91.9	0.82	0.77
B	Mirror	–	–	–	–	–	–
	Baffle	93.6	55.5	64.9	95.9	0.60	1.80
	BMG	–	–	–	–	–	–
C	Mirror	94.4	59.1	51.4	97.5	0.55	1.61
	Baffle	92.7	43.7	47.4	95.8	0.45	2.10
	BMG	92.2	42.4	35.9	96.4	0.39	2.20
D	Mirror	94.6	60.7	76.1	96.1	0.68	1.77
	Baffle	95.9	69.0	77.2	97.3	0.73	1.43
	BMG	95.8	68.4	78.5	97.2	0.73	1.41
Average	Mirror	93.8	64.6	71.2	95.7	0.70	1.40
	Baffle	93.4	59.4	70.9	94.9	0.64	1.56
	BMG	93.4	61.4	69.1	95.2	0.65	1.46

Average evaluation values of accuracy, precision, sensitivity, specificity, Dice coefficient and Hausdorff distance derived from cases A, C and D. An evaluation of case B created from baffle planner method, compared with the reference.

**Figure 12:**
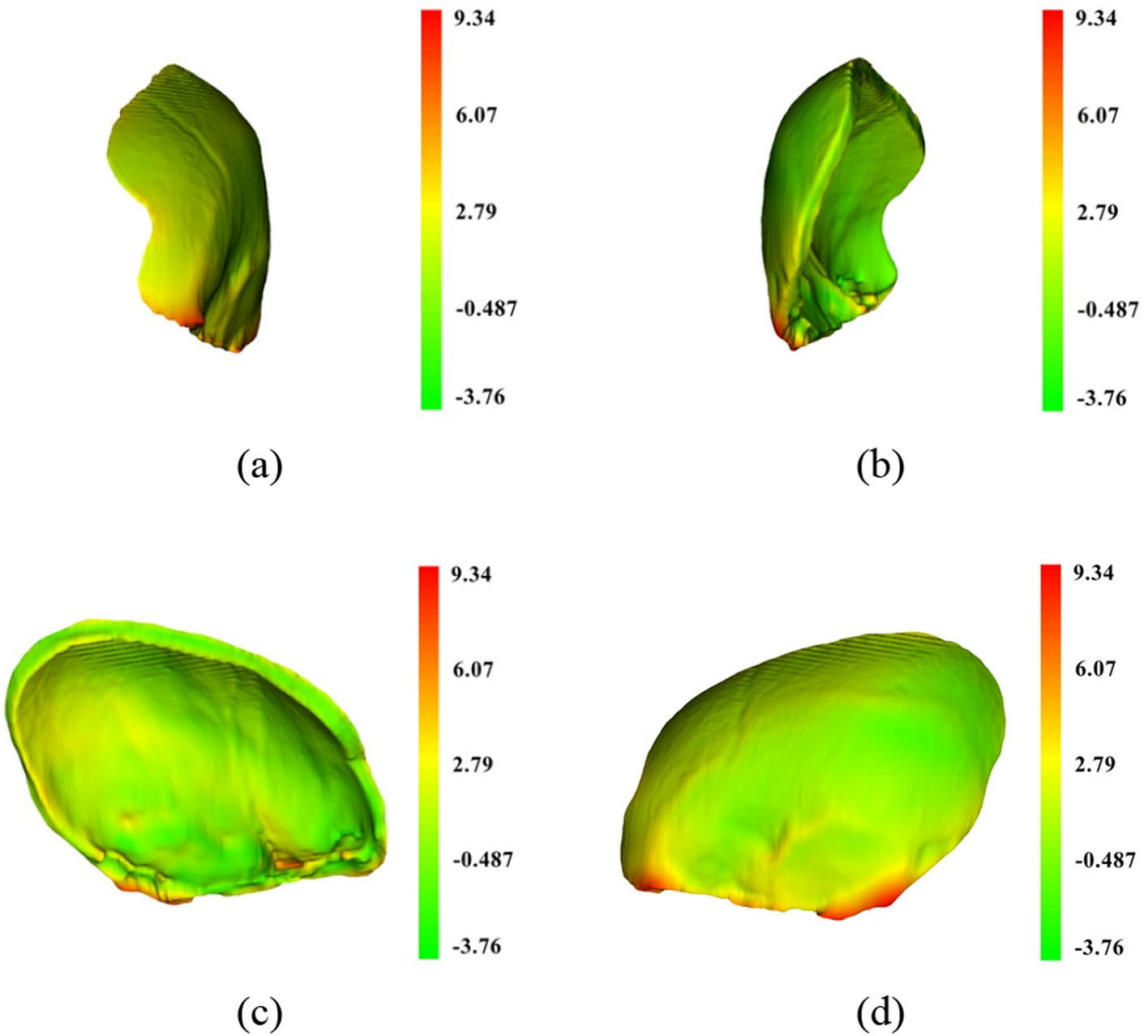
A comparison between the mirrored and reference models of case D using color deviation maps based on Hausdorff distance. The visualization (**a**) outer surface, (**b**) inner surface, (**c**) right side surface and (**d**) left side surface.

**Figure 13:**
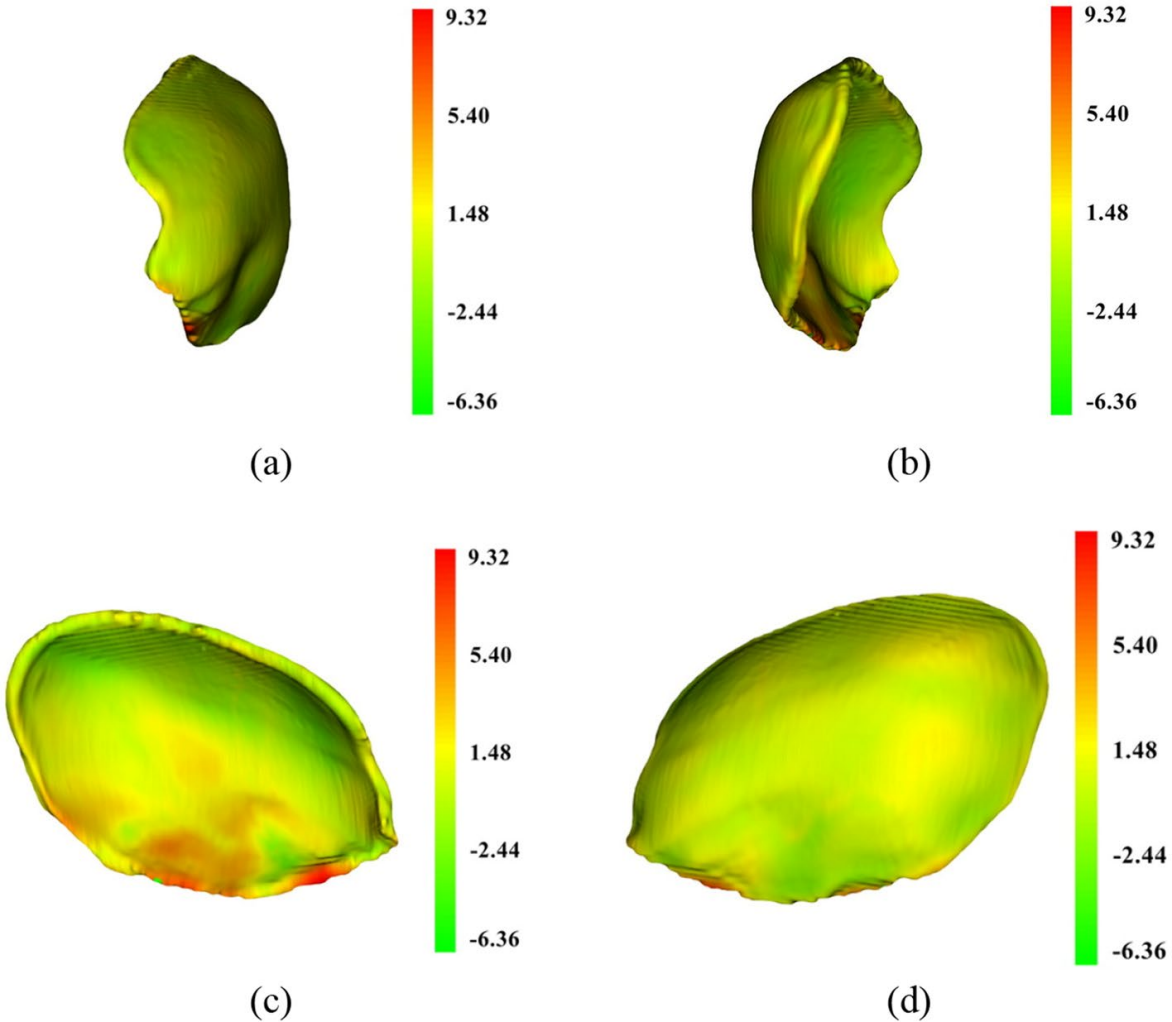
A comparison between baffle planner and the reference models of case D using color deviation maps based on Hausdorff distance. The visualization (**a**) outer surface. (**b**) Inner surface. (**c**) Right side surface. (**d**) Left side surface.

**Figure 14:**
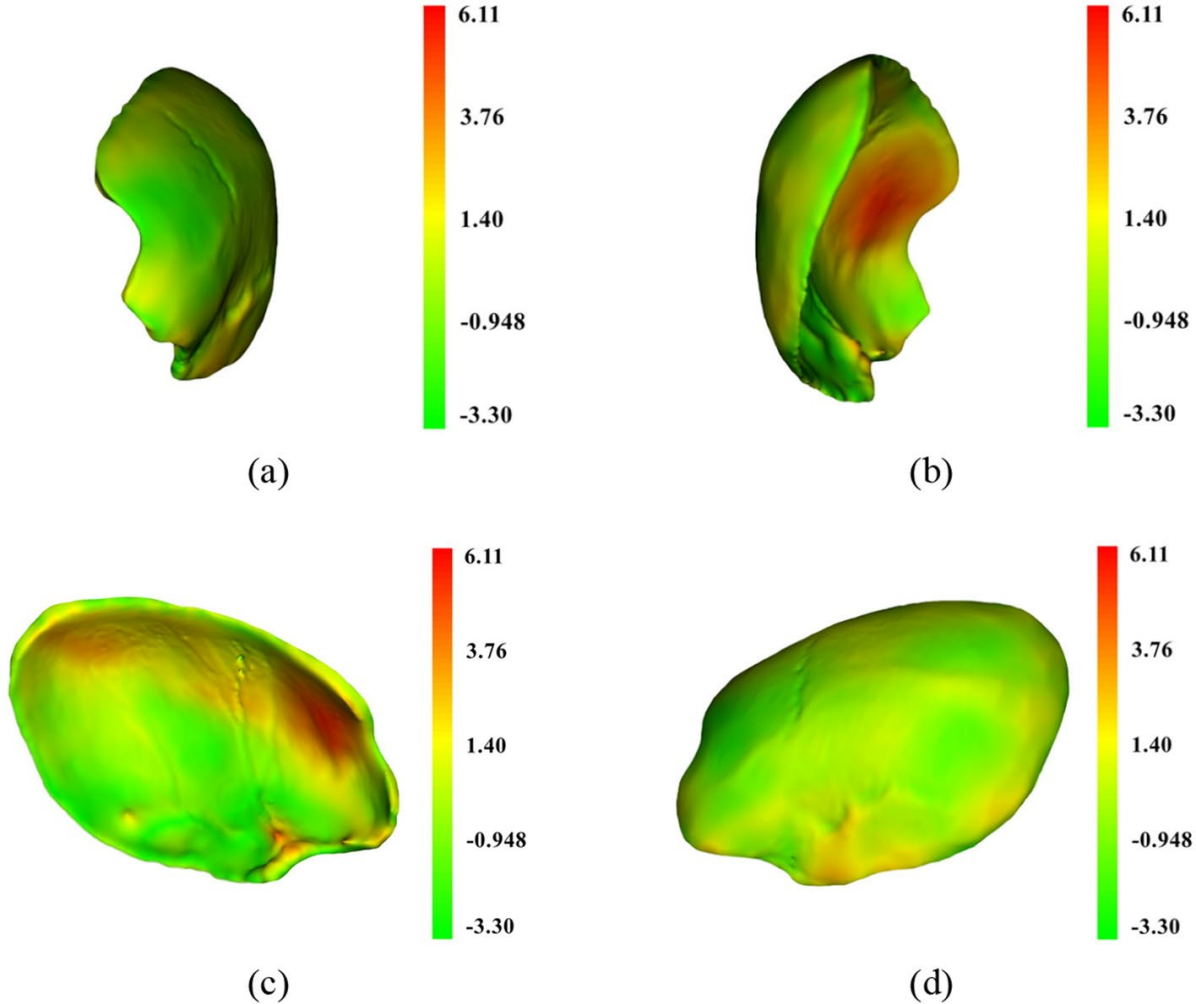
A comparison between the BMG and reference models of case D using color deviation maps based on Hausdorff distance. The visualization (**a**) outer surface. (**b**) Inner surface. (**c**) Right side surface. (**d**) Left side surface.

To visualize the difference between the designed and reference models, the shape-population viewer module in 3D Slicer was employed. Such viewer module represents the color shades of the model based on the Hausdorff distance described earlier. In the shade-population window, the area shaded in green, yellow, and red represents degrees of similarity between the designed and the reference models that ranges from negative deviation to positive deviation respectively. Figures 12, 13, 14 illustrate the degree of similarity of the three designed implant models (case D) created using the three techniques: mirror, baffle planner and baffle-based mirror guideline. The shade in red zone represents a larger volume of the designed model compared to that of the reference i.e., Hausdorff distance is positive. The shade in green zone represents a smaller volume of the modelled implants compared to that of the reference i.e., Hausdorff distance is negative. The yellow zone indicates the area of similarity between the designed and the reference models. It can be seen that most of the areas are in green and yellow meaning the size of the modelled implant is likely to be smaller than the reference models. Besides, it is remarked that the area around the edge of the models exhibits a larger size compared to that of the reference models.

According to Table 2, the volume of the designed implant models created using the three methods are generally larger than the reference implant models. This is because the designed implant models were created based on symmetrical skull thickness and similar defect volume, whereas thickness of the reference implants were designed to be thinner than the original skull. This is based on clinical experience of the neurosurgeons who prefer to reduce thickness of implants to prevent collision with swollen brain tissue. It is therefore noted that all performance metrics can reflect with warning. For example, the similarity index is not absolutely correlated with the size of the implant model, surface area error, volume error, Dice coefficient and Hausdorff distance. For instance, case D, which has a larger defect size than case C, shows better matching with the reference implant model.

The BMG method was aimed to provide curvature guidelines for the baffle method. Without such curvature guidance in baffle processing, it is difficult to draw or estimate appropriate 3D curves fit to defect seamlessly. This also requires expertise with experience, so that the BMG method is beneficial for non-midsagittal plane cases. For cross midsagittal plane cases, baffle method is still preferable. The simplified processing workflow is therefore instead the focus on this study. In future work, we plan to verify the designed implant models by printing and comparing them to the reference models. After this step is completed, we will conduct a clinical experiment using the proposed models.

## Conclusions

This study has presented and evaluated the three processing workflows for modelling craniofacial implant: mirror and baffle planner and baffle-based mirror guideline. We found that the mirror method was practical for a case that healthy skull region can be completely flipped to the defect region. However, without healthy reference skull, the mirror method was unable to model region across the midsagittal plane. As a result, an anatomical landmark remains a challenging issue. The baffle planner module offers a flexible prototype model fit independently to any defect location. However, the baffle planner method requires customized refinement of contour and thickness to fill the missing region seamlessly. The process also relies on a user’s experience and expertise. The baffle planner module offers an interactive tool for creating a 3D implant model regardless defect location. However, the baffle planner method requires manual adjustment of thickness and curvature to form the defect shape. The proposed baffle-based mirror guideline method exploits advantages from both approaches and presents improvement in case defect overlapping on left and right hemisphere. In future work, a precise method could be proposed by integrating an AI-based guideline system to automatically generate appropriate implant models for different defect scenarios.

## Supplementary Information

Supplementary Information 1.

Supplementary Information 2.

Supplementary Information 3.

Supplementary Information 4.

Supplementary Information 5.

## Data Availability

All data generated or analyzed during this study are included in this published article (and its supplementary information files).
