# Supplementary material for: The simplified tailor-made workflows for a 3D slicer-based craniofacial implant design
Source: Sci Rep. 2023 Feb 17;13:2850. doi: 10.1038/s41598-023-30117-w (PMC9938178; doi:10.1038/s41598-023-30117-w)
Supplement: Supplementary file 1 — Supplementary Information 1. [file 41598_2023_30117_MOESM1_ESM.pdf]

Appendix

The design of the implants was conducted using the three freeware: 3D Slicer, Meshmixer and Blender. The procedural steps of the implant design by the experienced neurosurgeon can be briefly described as follows:

(1) The 3D Slicer software is used to segment only skull. This is manually performed by applying a threshold value (in the Hounsfield unit) ranging between 100 and 400 to the original DICOM file. The appropriate threshold depends on the area being modeled. For example, for defects around the eye and nasal area, rich detail may be required, so that a low threshold value around 100 - 200 can be applied. However, if the defect occurs around the side or top of the skull, a higher threshold value between 300 – 400 is needed. After applying a suitable threshold value, the segmented skull model is saved as "skull.stl". Note that an appropriate threshold value is selected using a trial-and-error method and judged optically by the experienced neurosurgeon.

(2) Meshmixer is used to create the mirror area which can be described into 3 sub-steps:

(2.1) First open the "skull.stl" from step (1). Then, select the "Mirror" command in Meshmixer (under the edit menu), which will automatically create a mid-plane (i.e. the sagittal plane by default). The command "Mirror" allows the user to select indefectible side in which we name as "a mother-side", i.e., a source to mirror. After mirroring, the user will see both the mother-side and mirror-side indefectibly. Now the user deletes the mother-side and leaves only the mirror-side to be illustrated. Lastly, the user saves the mirror-side as "indefectible_side.stl" and closes the window to prepare for the next step.

(2.2) Open "skull.stl" from step (1) again. Then, select the "Mirror" command in Meshmixer to create another mid-plane. This time, choose the defect side as a mother-side (opposite to the selected side in step 2.1). Repeat the procedure as step 2.1 by deleting the mirror-side and then saving only the mother-side (defective side) as "defective_side.stl". In this step, the user must not close the current window and move to the next step.

(2.3) Open the "indefectible_side.stl" from step (2.1), which will show the indefectible side overlaid on the defective side. Now, the user can adjust the position of the indefectible side so that it overlaps the defect as much as possible. This is because the left and right sides of the skull may not be symmetrical. The use can set the pivot point of the indefectible side and visually observe when the edge of the defective side is closely fit. Then, the command "Boolean Subtraction" is used to obtain the implant model.

(2.4) Edit and decorate some parts by deleting residual area using the plane cut command. The thickness of implant is generally made thinner than the mirror side since the brain tissues could be swollen. However, the curvature is modified carefully to fit the skull.

Please note that in the past, the Blender software was used for subtraction process as step (2.3). However, it is now possible to do so using Meshmixer, as elaboration in step 2.3. Therefore, there is no longer a need to use Blender.

By implementing such steps using 3D Slicer and Meshmixer, we should note that only some experienced neurosurgeons who understand both the structural skull and 3D computer vision are able to proceed and follow those steps successfully. Furthermore, in terms of model editing, it is necessary to understand the representation of the 3D model, so that such steps (including the editing step) will not ruin the final implant model. Furthermore, after obtaining the implant model, onsite decoration (during operation) is practically needed since some detail of a prototype may not be aligned with the real skull as the 3D printing scale or the resolution of the printing may not be fine enough. Therefore, the traditional procedure based on the experienced neurosurgeon is in fact complicated and may be difficult to follow.
