# Supplementary figures and images for: The simplified tailor-made workflows for a 3D slicer-based craniofacial implant design
Source: Sci Rep. 2023 Feb 17;13:2850. doi: 10.1038/s41598-023-30117-w (PMC9938178; doi:10.1038/s41598-023-30117-w)

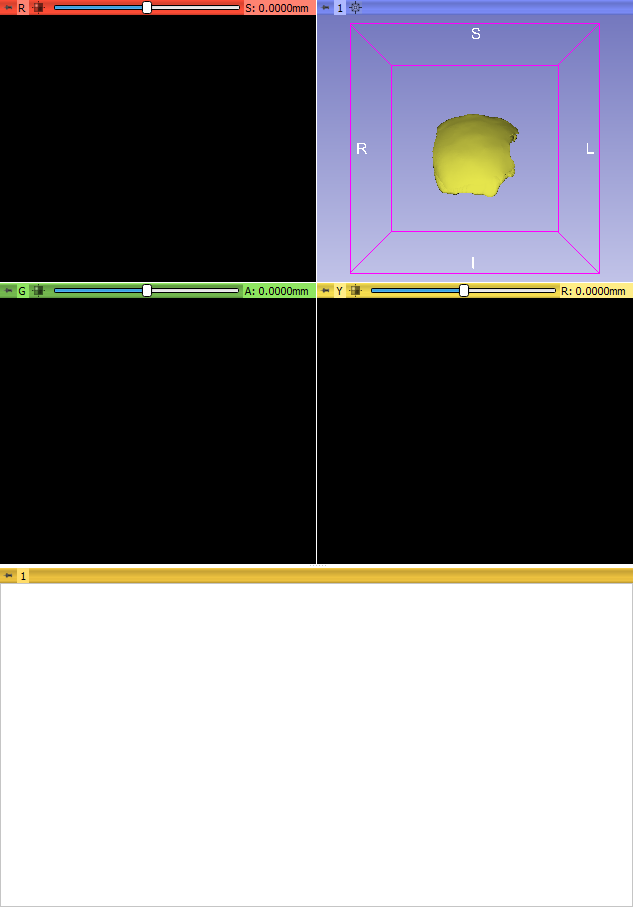

Supplement: Supplementary file 3 — Supplementary Information 3. [file 41598_2023_30117_MOESM3_ESM.zip › CaseB_center/2023-01-17-Scene.png]
